# Sex-specific stress and biobehavioral responses to human experimenters in rats

**DOI:** 10.3389/fnins.2022.965500

**Published:** 2022-07-22

**Authors:** Jamshid Faraji, Mirela Ambeskovic, Nevyn Sauter, Jaxson Toly, Kera Whitten, Nayara Antunes Lopes, David M. Olson, Gerlinde A. S. Metz

**Affiliations:** ^1^Department of Neuroscience, Canadian Centre for Behavioural Neuroscience, University of Lethbridge, Lethbridge, AB, Canada; ^2^Department of Obstetrics and Gynecology, University of Alberta, Edmonton, AB, Canada; ^3^Southern Alberta Genome Sciences Centre, University of Lethbridge, Lethbridge, AB, Canada

**Keywords:** experimenter sex, sex differences, cutaneous temperature, infrared thermography, oxytocin, corticosterone (CORT), stress response, hypothalamic-pituitary-adrenal (HPA) axis

## Abstract

Important factors influencing the outcome of animal experiments in preclinical research are often overlooked. In the current study, the reaction of female and male rats toward the biological sex of a human experimenter was investigated in terms of anxiety-like behaviors and physiological stress responses, as measured by infrared (IR) thermography, circulating corticosterone (CORT) and oxytocin levels. Female rats displayed consistently exacerbated anxiety-related behaviors along with elevated body surface temperature during repeated exposure to male experimenters. Experimental stress further intensified thermal responses to a male experimenter, especially in female rats. The behavioral responses to a male experimenter in females were associated with higher circulating CORT and lower oxytocin levels. Similar responses were induced by a T-shirt worn by a human male. The findings suggest that psychophysiological responses of female rats to a male experimenter are influenced by both visual and olfactory cues. The results emphasize the need to not only consider sex differences in experimental animals, but also standardize and report the experimenter’s biological sex to avoid ambiguity in the generation and interpretation of results.

## Introduction

Humans (Wagner et al., 2015) and non-human animals (Torquet et al., 2018) are impacted by interpersonal relationships. Laboratory practice with animals typically requires experimenters to build and maintain a close interaction with the subjects, a process that unavoidably involves handling, transferring animals from the home cage to experimental settings and performing other procedures. These aspects of manipulation may also interact with the animals’ inherent factors (e.g., sex, age, and species) and environmental influences (e.g., stress, nutrition, rearing and housing conditions) to create significant inconsistency in experimental results. Even when taking particular care about standardizing procedures, controlling for age, sex, litter effects, time of day and season, experimental research still creates situations where inter- and intra-rater concordance and experimenter-dependent manipulations affect the results (Rumenik et al., 1977; Rosenthal, 1980). Reasons for this failure include variations in laboratory procedures, the complex nature of research with live rodents, and transgenerational inheritance of ancestral experiences and epigenetic regulators of phenotypic traits (Zucchi et al., 2012).

Extensive research has documented that stress inevitably confounds measurements and interpretation of experimental results. Particularly the behavioral, physiological and metabolic domains are influenced by elevated activity of the hypothalamic-pituitary-adrenal (HPA) axis (Faraji and Metz, 2020; Faraji et al., 2020; Poplawski et al., 2020), which shows strong sexual dimorphisms and may even trickle down to subsequent generations (McCreary et al., 2016; Ambeskovic et al., 2019, 2020). Stress is the single most salient influence on experimental outcomes, affecting emotion, cognition, movement and sensory function, and underlying inflammatory and physiological response of all organ systems, including the brain (Metz, 2007). In general, female rodents seem to be more vulnerable than males to social and behavioral stressors in terms of circulating oxytocin (OT) responses (Faraji et al., 2018a), which represents a critical counterplayer to the stress response.

It was shown that rodents respond to male but not female human experimenters with a robust stress response and stress-induced analgesia (Sorge et al., 2014). In particular, mice of both sexes observed by male experimenters displayed lower levels of pain than those observed by females (Sorge et al., 2014). Hence, the sex of a human experimenter may not only influence behavioral and physiological measurements, but also the interpretation of potential sex differences in the data. The impact of an experimenter’s sex on scientific results in life sciences has become a matter of significant concern (Fanelli, 2018), and urged us to (1) address a significant gap in knowledge by exploring how an experimenter’s sex affects behavioral and physiological stress responses, and (2) extend previous findings (Sorge et al., 2014) in a rat model. Here, we used an array of established stress assessments in addition to infrared (IR) thermography, a non-invasive imaging technique of surface thermal changes linked to stress not requiring animal handling or manipulation (Tattersall, 2016; Faraji and Metz, 2020), regardless of HPA axis response. IR thermography provides an ideal tool for stress assessment because the peripheral autonomic nervous system, which regulates heart rate and breathing, tissue metabolism, respiration, and surface blood perfusion determines biological heat emission during short-term aversive experiences (Ioannou et al., 2014). In spite of its particular translational value, this technique has not yet been extensively used in laboratory rodents. The results show that female rats are more likely influenced by an experimenter’s sex than males, confirming the significance of a male observer effect in female behavior and physiology (Sorge et al., 2014). We show that olfactory and visual cues by a male experimenter activates the HPA axis with potentially wide-ranging and lasting effects on behavioral and physiological outcomes. The findings confirm earlier reports (Faraji et al., 2018a) of females being more vulnerable than males in terms of complementary corticosterone (CORT) and OT responses to social stimuli.

## Materials and methods

### Animals

Female and male Long Evans rats, 8–10 weeks old at the beginning of the experiment, were used in this study. The animals were housed in trios of the same sex in standard housing under a 12:12 h light/dark cycle with light starting at 07:30 h, and food and water provided *ad libitum*. The room temperature was set at 22°C, and experimental procedures were conducted during the light phase of the cycle at the same time of day. All animals were habituated in their assigned housing condition for 2 weeks before any experimental manipulation commenced. Aspen wood chips mixed with shredded paper bedding material was used in all home cages and changed once per week by a female animal care staff. Animal care personnel were recommended to minimize their physical contacts with animals while providing animal husbandry. Animals were briefly removed from their cages for 2–3 min when changing the bedding material was necessary. All experiments were carried out in compliance with ARRIVE guidelines and were approved by the University of Lethbridge Animal Care Committee in compliance with the standards set out by the Canadian Council for Animal Care (CCAC).

### Experimental design

**FIGURE 1:**
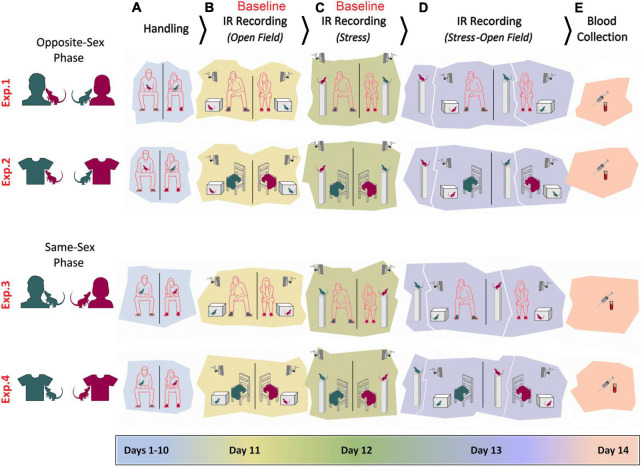
Experimental design. Female and male adult rats were exposed to female and male experimenters in four distinct experiments (Exp1–4). In Experiments 1–2, rats were exposed to opposite-sex experimenters or their T-shirts, whereas rats in the Experiments 3–4 were required to be handled and/or tested by same-sex experimenters. In all experiments animals were manipulated for handling **(A)**, pre-stress open field testing **(B)**, stress **(C)**, stress and post-stress open field testing **(D)**, and blood sampling **(E)**. Rats’ thermal responses to the experimental procedures were recorded by an infrared (IR) thermographic camera in the presence or absence of the experimenters.

Figures 1A–E illustrates the time course of experimental manipulations in four experiments. Opposite-sex phase (Experiment 1): The female rats (*n* = 6) were handled and tested by a male experimenter. The male rats (*n* = 6) were handled and tested by a female experimenter. Rats were handled daily for a total of 10 days (Days 1–10). Each rat was also handled for 2 min prior to any experimental manipulations on the four subsequent days. The experimenter remained in the room during testing, quietly positioned approximately 50 cm from the animal and testing apparatus (Days 11–13). Animals were handled by the same experimenters for 2 min per rat before being anesthetized with isoflurane for blood sampling (Day 14). Opposite-sex phase (Experiment 2): Experiment 2 was carried out in the same manner as of Experiment 1, but instead of the experimenter being present in the room, a cotton T-shirt was placed on a chair approximately 50 cm away from the animal (Sorge et al., 2014). The T-shirt was worn by the experimenter for at least 12 h prior to the assessments. The Same-sex phase (Experiment 3): All procedures were identical to Experiments 1 and 2, except that female rats (*n* = 6) were handled and tested by a female experimenter and male rats (*n* = 6) were handled and tested by a male experimenter. Same-sex phase (Experiment 4): All procedures were identical to Experiment 3 except that animals were tested in the presence of the experimenters’ T-shirt while the experimenters remained outside of the room for each assessment. Again, the T-shirts were previously worn by the experimenter for at least 12 h beforehand. For all testing procedures, rats were transported in their home cages to a designated test room. Female and male rats were tested and filmed in separate rooms.

### Handling

In all experiments, animals were handled individually for 5 min daily by their male or female experimenters for a total of 10 days prior to any experimental manipulations. The long-term handling protocol ensured habituation of the rats to the experimenters. The rats were handled in the housing room in a seated position on the lap of the experimenter for 2 min and then handled near the chest of the experimenter for an additional 3 min to facilitate olfactory habituation. Experimenters used their same own lab gown during all handling and test sessions.

### Open field task

The open field task (OFT) was used to assess anxiety-like behavior in rats. The apparatus consisted of a square box (50 × 50 cm) made of transparent Plexiglas and surrounded by walls (45 cm height). Each rat was individually placed at the center of the box and video recorded for 7 min with an IR thermographic camera mounted above the open field. The animals’ movements were analyzed for thigmotaxis (time spent close to the walls; 8 cm width) by an experimenter blind to experimental conditions. Thigmotactic behavior was analyzed as an indicator of anxiety and equi-emotional state (Fonio et al., 2012) in the open field (Faraji et al., 2020). After testing each animal, the apparatus was cleaned with 1% Virkon (Antec International Ltd., Suffolk, United Kingdom).

### Stress procedure

The elevated platform stress protocol used as a mild psychological stress was modified from that described previously (Mychasiuk et al., 2011). Animals were individually placed on the platform (20 cm × 20 cm on a Plexiglass stand 1 m above ground) for a single 7-min session in the morning hours. After the animal was placed on the platform and while it was filmed, the experimenter was quietly sitting (Experiments 1 and 3C), or the experimenter’s T-shirt was placed (Experiments 2 and 4C) on a chair a short distance (∼50 cm) away from the stand. Animals were removed from the platform and returned to the home cage by the experimenter when the stress session was terminated, and the platform was cleaned with 1% Virkon (Antec International Ltd., Suffolk, United Kingdom) between stress sessions.

### Infrared thermal imaging

The pre-stress thermal imaging was performed immediately after a 2-min handling session. A FLIR IR thermographic camera (FLIR T450sc, Sweden; fixed emissivity = 0.98 specified for skin in the manufacturer’s emissivity table) mounted on top of a transparent Plexiglas box or an elevated platform recorded the cutaneous temperature in rats. IR imaging occurred in a windowless room with a steady temperature set at 22°C and a relative humidity of ∼50%. Animals were protected from direct ventilation.

As previously reported (Faraji and Metz, 2020), animals were placed individually at the center of the Plexiglas box in a prone position, and the IR thermographic recording was performed above the box without lid because IR radiations are blocked by Plexiglas or stainless steel. The camera was placed ∼80 cm above the animal and was able to follow changes in the animal’s surface temperature and its immediate surrounding with thermal resolution of 320 × 240 pixels per image, thermal sensitivity of <30 mK at 30°C, and 60 Hz acquisition rate.

Infrared thermal profiles were then saved and analyzed using the FLIR image processing software (FLIR ResearchIR Max software 4.40.6.24). For the purpose of the thermal analysis, two principal regions of interest (ROI) were chosen. (1) Head (including eyes), covering a major portion of the frontal and parietal surfaces. An approximate measure of the sagittal suture allowed the elliptic ROI to split the top of the head up into left and right sides. Also, a small segment of interparietal bone was included in either right or left ROIs. To control the effect of the position of each animal on the emitted thermal irradiations, the best postural condition for the head was chosen when rats were moving with their head oriented straight ahead without deviation to the side. (2) Back, an oval-shape ROI included lower thoracic and upper and lower lumbar levels extended to the abdominal parts at equidistance from approximately 2.5 cm off the midline. In total, three ROIs (head [left and right] and back) were considered for analysis of changes in surface temperature. For sampling, 5–7 frames representing 5–7 time bins (one frame per each minute of the IR imaging) adjusted to the corresponding ROIs from the head and back were chosen for each animal. ROI sizes were identical for all frames and rats. The best-fit area to the ROIs in each frame/time bin was determined on the basis of the animal’s dorsal posture among nearly 1,750 single frames when approximately all relevant regions were bounded by the radius of the ellipses and/or when the animal was found in a prone position with all four limbs on the ground.

### Blood samples, corticosterone assessment, and oxytocin assay

Blood samples were collected in the morning hours between 9:00 and 11:00 am when the nadir for CORT typically occurs in nocturnal rodents. Blood samples were collected randomly within groups to reduce potential confounding effect of intrinsic factors such as social hierarchy. Briefly, rats were transported individually to the surgical suite and were handled by their assigned male or female experimenters for 2 min prior to anesthesia with 4% isoflurane. During 2–3 min of anesthesia, 0.5–0.9 ml of blood was collected from the tail vein using a heparinized butterfly catheter. Blood samples were taken by a separate male experimenter while the animals were anesthetized. Blood was then transferred to centrifuge tubes and plasma was obtained after centrifugation at 5,000 rpm for 5 min. The plasma samples were stored at −80°C until analyzed for CORT and OT concentrations. Plasma CORT levels were determined by enzyme-linked immunosorbent assay (ELISA) using commercial kits (Cayman Chemical, Ann Arbor, MI, United States). Plasma OT levels were determined by enzyme immunoassay (EIA) using a Human/Mouse/Rat Kit (RayBiotech Inc., Norcross, GA, United States) according to the manufacturer’s protocol. The OT concentration (ng/ml) was determined by plotting the mean absorbance of each unknown sample on the standard curve (range 0.1–1,000 ng/ml). The minimum detectable concentrations of OT was 3.6 ng/ml. The Intra- and inter-assay coefficient of variation (CV) were <10 and <15%, respectively, as reported by the manufacturer.

### Statistical analysis

Effects of main factors (Experiment–four levels; Rat Sex–two levels) were analyzed as independent variables for the thigmotaxis in the open field and the surface temperature in different ROIs and frames as dependent variables by repeated measure, one- and multivariate ANOVAs. *Post hoc* Tukey test was used to adjust for multiple comparisons when multi-level factors (e.g., experiments and frames) were needed to be compared. Familywise error was considered prior to the multiple *post hoc* analyses, if necessary. Also, to evaluate the magnitudes of the effects of experimental manipulations (here, elevated platform stress and experimenter sex in C-Baseline and D phases) on thigmotaxis, body surface temperature, effect sizes (η^2^ for ANOVA) were calculated. Values of η^2^ = 0.14, 0.06, and 0.01 were considered for large, medium, and small effects, respectively. The individual experimenters or T-shirts in all experiments did not differ significantly from one another within-sex. Moreover, because the plasma CORT and OT values in the present study were not normally distributed, the Mann–Whitney *U*, a rank-based non-parametric test was used to compare means of the two groups (females vs. males) for a single dependent variable, either CORT or OT. Correlations between variables (surface temperature, thigmotaxis, CORT, and OT) were analyzed by Pearson product-moment and Spearman rank-order correlation coefficients. One to two data points identified as influential statistical outliers were excluded from correlational analysis to prevent the impact of outliers on the values of correlation coefficient or regression relationships. Two data points were also excluded from the CORT and OT data prior to analysis. In all statistical analyses (IBM SPSS statistics, Version 21, United States), a *p*-value of <0.05 (two-tailed) was chosen as the significance level. Results are presented as mean ± standard error.

## Results

### Baseline: Female and male rats differently respond to a male experimenter by anxiety-related behaviors

**FIGURE 2:**
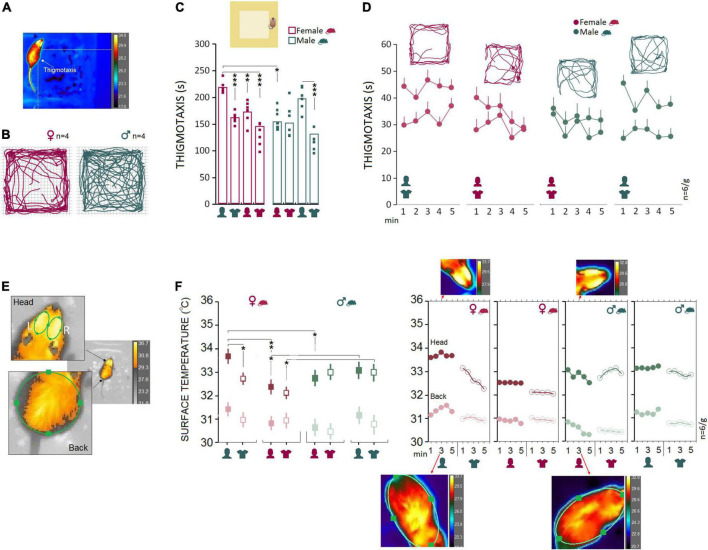
Open field testing and IR imaging. **(A)** Exploratory movements of female and male rats were analyzed for thigmotaxis or the repetitive pattern of exploration near to the wall in the open field. **(B–D)** Thigmotaxis in Experiment 1 was significantly impacted when female rats were required to explore the open field in the presence of an opposite-sex experimenter. The multipath shown in the panel **(B)** motion track graphics compares thigmotaxis taken from four representative rats during Exp1. Small squares represent individual rats in each group and experimental session. **(E)** The IR thermographic imaging showing two regions of interest [head (left and right) and back] in assessments of surface temperature during the open field exploration. **(F)** Because there were no differences in the thermal responses between the left and right sides of the head, the average of cutaneous temperatures for the left and right sides were used. Females showed consistently higher cutaneous thermal temperatures than males for the head and back (left panel). The inset IR graphic output (right panel) provide samples of thermal differences in females and males in both ROIs. However, when animals were tested by same-sex experimenters in Experiments 3 and 4, male rats showed higher thermal responses than females in the head. *p ≤ 0.05, **p ≤ 0.01, ***p ≤ 0.001; one-way and repeated-measure ANOVA, *n* = 6/group. Error bars show ± SEM.

Female rats (*n* = 6) responded to the presence of the male experimenter by more pronounced thigmotactic behaviors and higher variations in the surface thermal outcomes than male rats (*n* = 6). Figures 2A,B illustrate the thigmotaxis area in the open field which comprised the marginal part of the open zone around the wall and the animals’ paths in the open field. Repeated measure ANOVA showed a significant effect of Group (rat sex; *F*_1_,_40_ = 13.55, *p* < 0.001, η^2^ = 0.25), Experimenter (*F*_3_,_40_ = 15.61, *p* < 0.000, η^2^ = 0.53) and interaction between Group and Experimenter (*F*_3_,_40_ = 7.96, *p* < 0.000, η^2^ = 0.37) when thigmotaxis was considered. Within-group comparison indicated that thigmotaxis in female rats significantly increased in the presence of a male experimenter (Experiment 1) compared to other experimental conditions (all *p* < 0.01, *Post hoc* Tukey; Figure 2C). However, thigmotaxis in male rats increased only in the presence of a male experimenter when compared to a male’s T-shirt (*p* < 0.000, *Post hoc* Tukey; Figure 2C). Also, thigmotaxis in females significantly increased in Experiment 1 when they were required to explore the open field in the presence of a male experimenter compared to males in the presence of a female experimenter (219.34 ± 5.49 vs. 154.33 ± 5.49 s; *F*_1_,_10_ = 69.97, *p* < 0.001, η^2^ = 0.87; Repeated-Measure ANOVA). We then tested whether this effect could be replicated with T-shirts worn by men in Experiment 2, and in Experiments 3 and 4 in which animals were exposed to the same-sex experimenters and their worn T-shirts. Thigmotaxis in Experiments 2–4, however, revealed no significant differences between females and males (all *p* ≥ 0.05; Figures 2C,D). Hence, mainly the equi-emotional aspects of exploration were impacted by the presence of the opposite-sex experimenter only in females. Figure 2E shows thermographic images of two ROI [head (left and right) and back] in assessments of surface temperature during open-field exploration. Females and males (*n* = 6/group) displayed different patterns of changes in cutaneous temperatures across all five time bins. Also, the heat change pattern in the ROIs appeared to follow an order of head > back where the head in both groups emitted more heat (females, 33.67 ± 0.27°C vs. males, 32.78 ± 0.27°C) than back (females, 31.34 ± 0.09°C vs. males, 30.51 ± 0.09°C).

Results showing the thermal changes during open field exploration in all experiments are displayed in Figure 2F. Repeated measure ANOVA showed no effect of Group (*p* ≥ 0.05), but a main effect of Experimenter (*F*_3_,_40_ = 3.96, *p* < 0.01, η^2^ = 0.22) and interaction between Group and Experimenter (*F*_3_,_40_ = 10.88, *p* < 0.000, η^2^ = 0.40). Female rats emitted more heat in the head in the presence of a male experimenter compared with their exposure to a female experimenter (33.68 ± 0.24°C vs. 32.50 ± 0.24°C; *p* < 0.01; *Post hoc* Tukey; Figure 2F, left panel). No significant difference was observed in male rats when thermal responses to female and male experimenters were analyzed (all *p* ≥ 0.05). Also, the observed interaction between the rat and experimenter allowed us to compare within female and male rats vs. female and male experimenters. The heat emitted from head and back ROIs presented a robust effect of rat sex when animals were required to explore the open field in the presence of the opposite-sex experimenters in Experiment 1. Similar to thigmotaxis, compared to males the cutaneous thermal changes in females revealed significantly higher temperatures in the head (*F*_1_,_10_ = 8.05, *p* < 0.05, η^2^ = 0.44) and back (*F*_1_,_10_ = 39.82, *p* < 0.001, η^2^ = 0.79). No effects of Side (left vs. right) and Time Bin were found. Also, there was no sex difference in thermal responses to the T-shirts worn by the opposite-sex experimenter in Experiment 2. We thus failed to replicate the sex-specific effect of the male observer on the cutaneous thermal levels in Experiment 2 when animals were only exposed to the T-shirts worn by the opposite-sex experimenters.

However, the profile of thermal changes in Experiments 3 and 4 (same-sex phase) was different among females and males when animals were exposed to the same-sex experimenters and their T-shirts. In both experiments, male rats displayed an exacerbated thermal response in the head only to the male experimenter (Experiment 3; males, 33.15 ± 0.18°C vs. females, 32.51 ± 0.18°C) and the experimenter’s T-shirt (Experiment 4; males, 32.91 ± 0.23°C vs. females, 32.08 ± 0.24°C) relative to females. MANOVA conducted for the head did show significant effects of Rat sex (Experiment 3: *F*_1_,_10_ = 6.15, *p* < 0.05, η^2^ = 0.38; Experiment 4: *F*_1_,_10_ = 6.07, *p* < 0.05, η^2^ = 0.37), but Side (left vs. right), Back, and Time Bin (all *p* ≥ 0.05, Figure 2F, left panel). Thus, we confirmed that both female and male rats were vulnerable to a male experimenter. Females, however, displayed more exaggerated responses to the presence of the opposite-sex experimenter as shown by the increased thigmotactic behaviors and the surface temperature in the head and back.

### Baseline: Male rats are more susceptible than females to the presence of a male experimenter during a transient stressful experience

**FIGURE 3:**
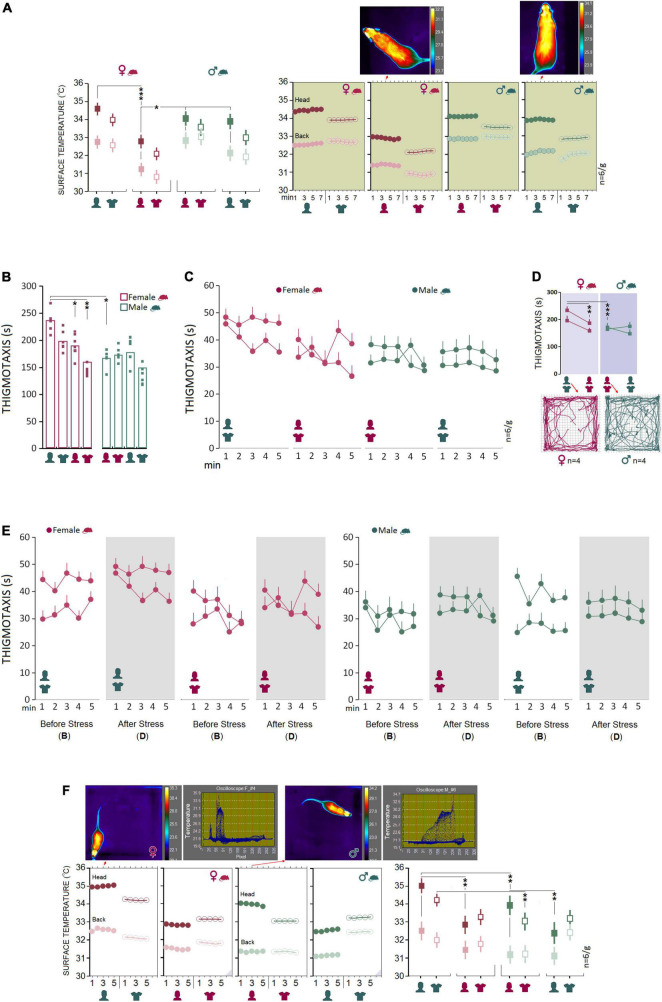
Stress and post-stress open field exploration. **(A)** No significant difference was observed between females and males in the surface temperature when rats were stressed in the presence of opposite-sex experimenters or their T-shirts. Inset thermal graphics compare changes in cutaneous temperature in two representative rats in min 4. **(B–D)** Although thermal responses during stress in Experiment 1 showed no sex differences, post-stress thigmotaxis noticeably increased in females. The multipath taken from four representative rats in each group is shown in the inset motion track graphics. **(E)** A comparison of the pre- and post-stress thigmotaxis indicated females spent more times near to the wall than males at both time points in the presence of a male experimenter (left panel). **(F)** Interestingly, females displayed higher thermal changes than males in the head and back when exposed to a male experimenter and the male’s T-shirt (Exp1 and 2) after stress. The inset thermal graphics represent thermal changes in min 3 in a female and male rat accompanied by the pertinent oscilloscopes. Purple triangles in the panels represent *D.* IR recording (stress-open field). *p ≤ 0.05, **p ≤ 0.01, ***p ≤ 0.001; one-way and repeated-measure ANOVA, *n* = 6/group. Error bars show ± SEM.

Experimental procedures typically impose mild to severe levels of unintended stress upon rodents in laboratory practice and may confound the outcomes of the manipulations. Here we examined how rats that previously experienced stress respond to human experimenters of the same or opposite sex. We chose a single-session psychological stress procedure in the presence of the opposite- and same-sex experimenters and their T-shirts for 7 min (day 12). A comparable pattern of thermal changes was found in females and males in Experiments 1 and 2 when animals did experience stress in the presence of the opposite-sex experimenters or their T-shirts (Figure 3A). Repeated measure ANOVA indicated a significant main effect of Group (*F*_1_,_40_ = 27.79, *p* < 0.001, η^2^ = 0.41), Experimenter (*F*_3_,_40_ = 59.57, *p* < 0.001, η^2^ = 0.41) and interaction between the Group and Experimenter (*F*_3_,_40_ = 4.93, *p* < 0.005, η^2^ = 0.27) when thermal responses during stress were considered. No significant effect of Time Bin was observed (all *p* ≥ 0.05). Female rats emitted greater heat during stress in the presence of male experimenters than female experimenters (Head: 34.43 ± 0.01°C vs. 32.88 ± 0.02°C; *p* < 0.001; Back: 32.53 ± 0.01°C vs. 31.39 ± 0.03°C; *p* < 0.001; *Post hoc* Tukey). No significant differences were observed in male rats’ thermal responses to the transient stress (Figure 3A, left panel). Also, thermal activity in response to the same-sex experimenter and their T-shirts in Experiments 3 and 4 revealed a male-specific response to the male experimenter. Male rats showed higher thermal responses than female rats in both head (33.87 ± 0.28°C vs. 32.87 ± 0.28°C; *F*_1_,_10_ = 5.94, *p* < 0.05, η^2^ = 0.37; ANOVA) and back (32.09 ± 0.16°C vs. 31.39 ± 0.16°C; *F*_1_,_10_ = 8.57, *p* < 0.01, η^2^ = 0.46; ANOVA) when stressed in the presence of a female experimenter. When exposed to the T-shirts worn by the same-sex experimenter, however, only heat emitted from back in males indicated a significant difference from females (31.93 ± 0.22°C vs. 30.88 ± 0.22°C; *F*_1_,_10_ = 10.89, *p* < 0.01, η^2^ = 0.52; Figure 3A). Thus, it appears that male rats experience greater IR thermal changes than females when exposed to a male experimenter during stress.

### Stress exerts sex-specific effects on affective state and intensifies thermal responses to a male experimenter, especially in female rats

To assess the interactions between the stress paradigm and the presence of an experimenter, animals underwent stress in the absence of an experimenter. Immediately after the stress, animals were tested and filmed for IR thermography in the open field in the presence of an experimenter. Thigmotaxis influenced by a 5-min session of elevated platform stress is illustrated in Figures 3B–D.

Again, between-subjects effects by repeated measure ANOVA for post-stress thigmotaxis indicated a main effect of Group (*F*_1_,_40_ = 31.00, *p* < 0.001, η^2^ = 0.43), Experimenter (*F*_3_,_40_ = 12.88, *p* < 0.001, η^2^ = 0.49) and a significant interaction between Group and Experimenter (*F*_3_,_40_ = 6.74, *p* < 0.001, η^2^ = 0.33), where females significantly displayed more thigmotaxis in Experiment 1 than other conditions (all *p* < 0.05, *Post hoc* Tukey). No differences in thigmotaxis between experimental conditions were found in male rats. Also, thigmotaxis in Experiment 1 revealed that females spent significantly more time in the thigmotaxis area close to the wall than males in the presence of an opposite-sex experimenter (235.46 ± 7.31 vs. 165.85 ± 7.31 s; *F*_1_,_10_ = 45.28, *p* < 0.001, η^2^ = 0.81; one-way ANOVA). Further, a comparison between pre- and post-stress thigmotaxis showed no differences between female and male rats (all *p* ≥ 0.05, Figure 3E) indicating that the transient stress paradigm did not change anxiety-like behavior in the open field.

Analysis of the IR thermal responses to the experimenter by repeated measure ANOVA showed no significant main effect of Group (*p* ≥ 0.05), but an effect of Experimenter (*F*_3_,_40_ = 4.12 *p* < 0.01, η^2^ = 0.23) and significant interaction between Group and Experimenter (*F*_3_,_40_ = 3.47, *p* < 0.001, η^2^ = 0.47) *via* which both female and male rats experienced the same rate of thermal changes in the head in the presence of the opposite-sex experimenter as opposed to the same-sex experimenter (all *p* < 0.05, *Post hoc* Tukey; Figure 3F, right panel). Similar to the baseline thermal responses, however, female rats responded to the male experimenter and the male-worn T-shirt with an exacerbated cutaneous thermal reaction when compared with male rats after stress.

Further, the heat emitted from head (34.98 ± 0.18°C vs. 33.95 ± 0.18°C; *F*_1_,_10_ = 14.87, *p* < 0.01, η^2^ = 0.59; ANOVA) and back (32.55 ± 0.26°C vs. 31.33 ± 0.26°C; *F*_1_,_10_ = 10.59, *p* < 0.001, η^2^ = 0.51; one-way ANOVA) presented a robust effect of group (rat sex) in Experiment 1 where the cutaneous thermal responses to the male experimenter was significantly more exaggerated in female than male rats. Thermal changes in Experiment 2 also were higher in females than males in the head (34.18 ± 0.25°C vs. 33.04 ± 0.25°C; *F*_1_,_10_ = 10.44, *p* < 0.01, η^2^ = 0.51; one-way ANOVA) in response to the T-shirt worn by male experimenters. The significant effect of group, however, disappeared in Experiments 3 and 4 when animals were required to explore the open field in the presence of the same-sex experimenters and the experimenters’ T-shirts (all *p* ≥ 0.05; Figure 3F, right panel). No effect of Time bin was observed across all experiments (all *p* ≥ 0.05).

**FIGURE 4:**
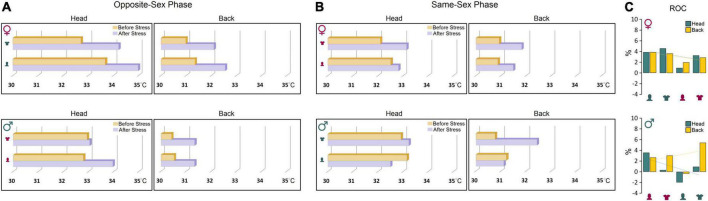
**(A,B)** Thermal changes prior to and after stress. Both groups were susceptible to the stress procedure in the presence of opposite-sex experimenters. However, female thermal responses in both ROIs were noticeably different from males before and after stress indicating higher vulnerability of females to the impact of stress and experimenter sex. **(C)** The rate of changes (ROC) provides further support for exacerbated thermal responses in female rats to experimenter sex after stress relative to male rats. Note the trendlines of thermal changes in the head and back in both sexes that noticeably depict sex-specific differences in the regional thermal responses to stress and experimenter sex.

Further comparisons of thermal changes prior to and after stress showed that both groups were susceptible to the stress procedure when responding to the opposite-sex experimenters (Figures 4A,B). However, the profile of thermal changes before and after stress in both ROIs in females was noticeably different from males indicating higher vulnerability of female thermal responses to the experimenter’s sex. Females’ susceptibility to the transient stress and the experimenter sex was also supported by additional analysis of the rate of changes (ROC) showing that female rats experienced larger changes in cutaneous temperatures after stress in response to experimenter sex [Female (head): 12.65% vs. Male (head): 2.83%; Female (back): 12.31% vs. Male (back): 10.78%, Figure 4C]. Thus, although behavioral responses to the experimenter sex remained unchanged after stress, the cutaneous thermal responses to both, opposite- and same-sex experimenters in females were noticeably affected by the transient stress.

### Corticosterone and oxytocin: Presence of male experimenters or their T-shirts was associated with elevated plasma CORT levels and reduced oxytocin levels in female rats

**FIGURE 5:**
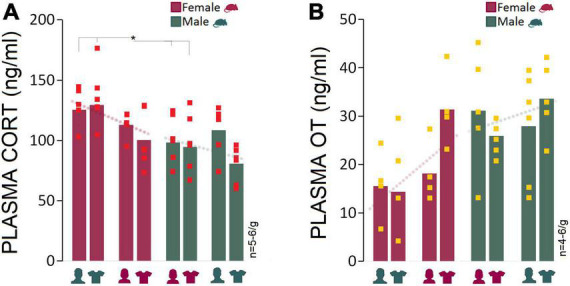
Corticosterone and oxytocin responses to stress and experimenter sex. **(A)** Average CORT levels in females across all experiments were higher than males. Exposure to an opposite-sex experimenter significantly elevated plasma CORT levels in female rats only. There were no significant differences between females and males in Experiments 3 and 4 when exposed to same-sex experimenters or their T-shirts. Red squares represent individual animals in each group. Gray boxes represent statistically significant differences between sexes (*p ≤ 0.05, Mann–Whitney *U*; *n* = 5–6/group). **(B)** Female rats responded to the presence of a male experimenter with reduced plasma OT levels. In contrast, male rats experienced marginal changes in plasma OT levels across experiments (*n* = 4–6/group).

The male experimenters and male-worn T-shirts induced sex-specific behavioral and neurophysiological changes that can be linked to higher anxiety-like behaviors and stress responses in rats. Here, we examined stress-induced activation of the HPA axis in circulating plasma CORT changes linked to experimenter sex. One female and one male rat were excluded from the CORT analysis due to technical issues. Generally, females had higher plasma CORT levels than males in the presence of a male experimenter (Mean Rank: 8.83 vs. 4.17; *U*_6,6_ = 4.000, *Z* = −2.24, *p* < 0.05; Mann–Whitney *U*) or their worn T-shirts (Mean Rank: 8.67 vs. 4.33; *U*_6,6_ = 5.000, *Z* = −2.08, *p* < 0.05; Mann–Whitney *U*) across the four experiments. The between-group differences disappeared in Experiment 3 when animals were exposed to a same-sex experimenter (Mean Rank: 6.00 vs. 6.00; *U*_5,6_ = 15.000, *Z* = 0.00, *p* = 1.000; Mann–Whitney *U*) or their worn T-shirts (Mean Rank: 7.92 vs. 5.08; *U*_6,6_ = 9.500, *Z* = −1.36, *p* = 0.17; Mann–Whitney *U*), although CORT values in females were still higher than those in males (Figure 5A). Overall, the HPA axis response to the experimenter was limited to female rats only in the presence of male experimenter or T-shirts worn by men.

We also hypothesized that elevated CORT induced by a male experimenter may dampen oxytocinergic influences that are linked to positive social interactions. The presence of a male experimenter was associated with a moderately reduced level of plasma OT in females (15.53 ng/ml) compared to males (31.15 ng/ml) in Experiment 1 (Mean Rank: 3.25 vs. 6.40; *U*_4,5_ = 3.000, *Z* = −1.71, *p* = 0.08; Mann–Whitney *U*) and experiment 2 (14.37 vs. 25.93 ng/ml, Mean Rank: 4.00 vs. 7.67; *U*_5,6_ = 5.000, *Z* = −1.84, *p* = 0.06; Mann–Whitney *U*) when female rats were exposed to men-worn T-shirts. Despite the decline in female OT levels in the presence of a male experimenter, the consistently higher OT levels in male rats across experiments indicated that the experimenter sex only had a marginal impact on the males’ oxytocinergic responses (Figure 5B).

### Correlational analysis: Behavioural responses to stress and the experimenter sex do not predict thermal changes

Because there were no hemispheric differences, the average of cutaneous temperatures for the left and right sides of the head were used for correlational analyses. Pearson’s correlation analysis revealed no significant relationship between thigmotactic behaviors in the open field and changes in surface temperature in the corresponding ROIs *before stress* (Supplementary Figure 1A), except for the back when females were exposed to a male experimenter (Experiment 1, *r* = −0.93, *p* = 0.01). Also, the only significant correlation between thigmotaxis and thermal responses after stress was observed in Experiment 1 for females in the head (Experiment 1, *r* = −0.90, *p* < 0.01; Supplementary Figure 1B). Thus, in most cases changes in thigmotaxis in the open field, either before or after stress, did not predict surface temperature in response to opposite- and the same-sex experimenters. Supplementary Figure 1C also illustrates correlations between the heat emitted during stress (C-Baseline) and post-stress exploration in the open field (D). Changes in surface temperatures during stress only predicted surface temperatures for the back in males when exposed to a T-shirt worn by a female (Experiment 2, *r* = 0.87, *p* < 0.05) and to a male experimenter (Experiment 3, *r* = −0.87, *p* < 0.05). In females, however, the correlations between the pre- and post-stress cutaneous temperatures only were significant in the head when they were exposed to T-shirts worn by female experimenters (Experiment 4, *r* = 0.89, *p* < 0.01) suggesting that stress-induced thermal changes did not reliably predict post-stress thermal responses to the experimenter sex in both groups.

Moreover, Spearman’s rho correlation coefficient showed that increased plasma CORT can reliably predict increased thigmotactic behavior indicating that the experimenter sex mediates both the HPA-axis activity and anxiety-like behavior, particularly in female rats (Supplementary Figure 2A). The observed correlation in females however was stronger than male rats in Experiments 2 (*r*_*s*_ = 1.000, *p* < 0.001 vs. *r*_*s*_ = 0.42, *p* = 0.397, *n* = 4–6/g) and 3 (*r*_*s*_ = 1.000, *p* < 0.001 vs. *r*_*s*_ = 0.94, *p* < 0.01, *n* = 4–6/group). In contrast, there was no correlation between CORT and surface temperature across experiments suggesting that in both sexes there are likely two distinct neurohormonal pathways that may determine the thermal and the HPA-related responses to the experimenter sex (Supplementary Figure 2B). Further analysis also revealed a significant negative correlation between CORT and OT levels exclusively in females in Experiment 1 (*r*_*s*_ = −1.000, *p* < 0.01, *n* = 4; Spearman’s rho) and 2 (*r*_*s*_ = −1.000, *p* < 0.01, *n* = 5; Spearman’s rho) where increased CORT levels were associated with reduced OT levels in the presence of a male experimenter or male-worn T-shirts. No significant correlations were found between CORT and OT levels in Experiments 3 (*r*_*s*_ = −0.667, *p* = 0.22, *n* = 5; Spearman’s rho) and 4 (*r*_*s*_ = −0.800, *p* = 0.20, *n* = 4; Spearman’s rho) in females (Supplementary Figure 2C).

## Discussion

The replication crisis represents a significant threat to the advancement of the life sciences and medicine (Van IJzendoorn and Bakermans-Kranenburg, 2021). The failure to reproduce results requires urgent attention to identifying the variables that can limit reproducibility of research in animal models. The impact of the sex of the experimenter on behavioral and biological processes in laboratory rodents is poorly understood and generally underestimated. Here we show that female and male rats display robust behavioral and physiological stress responses to the sex of the experimenter, a finding that mainly agrees with earlier data (Sorge et al., 2014). A human male experimenter induced more variations in female rats with changes that are linked to the dysregulation of the HPA axis and psychophysiological distress. These fundamental changes in the stress response may significantly influence the results of basic science and preclinical studies, emphasizing the need for consideration in experimental design, reporting of the research, and data interpretation.

Activation of the HPA axis represents a neurohormonal hallmark of response to stress in humans and animals (Metz, 2007; Faraji et al., 2018b), and its activity is characterized by prominent sex differences. In fact, females initiate the HPA-axis activity more rapidly in response to stressful stimuli and produce a greater output of stress hormones (Goel et al., 2014; Heck and Handa, 2019; Kokras et al., 2019). It appears that sex differences in animal research are not displayed consistently. A growing body of evidence show that sex differences in rodents stem, at least in part, from organizational effects of sex hormones (Burkitt et al., 2007), rearing/housing conditions (Juraska et al., 1984), and experimental protocols and parameters (Faraji et al., 2010). Hence, the present findings shows that the neurohormonal differences in the rats’ response to the experimenter sex that appear most pronounced in an opposite-sex framework. Therefore, animal studies involving male experimenters will more likely suffer from experimental bias.

Infrared thermal measures provide another sensitive indicator of the stress response, which were also impacted by experimenter sex. This observation reflects the sex-specific nature of thermoregulatory activity in rats (Faraji and Metz, 2020). Indeed, the peripheral autonomic nervous system that regulates perspiration and surface blood perfusion, predominantly determines heat patterns and gradients during aversive experiences (Ioannou et al., 2014). Furthermore, HPA-system activity alone does not fully encompass a complete picture of sex differences during psychophysiological disturbances as neuroendocrine stress-related responses in female rodents are different than males (Goel et al., 2014). Thus, cutaneous temperature variations may serve as an alternative physiological marker for stress, fear, tension, and anxiety (Engert et al., 2014; Lecorps et al., 2016; Gjendal et al., 2018). Here, females displayed greater levels of IR thermal susceptibility to what became recently known as the “male observer” effect (Sorge et al., 2014) when briefly stressed, even though the stress paradigm used in the current study was not salient enough to experimentally induce a prominent stress response in rats. This supports our earlier findings in mice that (1) thermal responses function partially independently of CORT levels, (2) may be more sensitive to subtle effects of stress, and (3) show greater effects in females (Faraji and Metz, 2020). The findings suggest a distinct neuroregulatory system in thermal response to psychologically threatening stimuli in rats. The present findings also show that synergy between a stressful stimulus and the presence of a male experimenter may further sway experimental outcomes. The experimenter sex may account for a significant portion of the variance of behavioral and physiological observations particularly in female animals.

In regard to the sex-biased thermal alterations and the OT inhibition in response to experimenter sex two mechanistic possibilities can be hypothesized. First, changes in the peripheral temperature in females that are modulated by central subsystems are sex-specific hormone-dependent responses mainly influenced by estrogens (Charkoudian and Stachenfeld, 2014; Charkoudian et al., 2017). In parallel with its thermal consequences, estrogen is also expected to centrally increase OT signaling (McCarthy, 1995), which eases the anxiolytic oxytocinergic function. This OT-mediated signal typically reduces the inhibition inherent to social encounters. The second possibility, alternatively, points to the inhibitory effect of glucocorticoid action on the OT function (Durlo et al., 2004; Vilela and Giusti-Paiva, 2011) which is influenced by sex steroids, especially estrogens (Heck and Handa, 2019). OT normally enhances the glucocorticoid response to acute aversive experiences (Neumann, 2002; Figueiredo et al., 2007). However, increased circulating CORT levels due to stress may reversely inhibit OT-mediated anxiolytic influences (Liberzon and Young, 1997; Neumann, 2002) depending upon the stress regimen and intensity. For instance, a prolonged stressful experience can reduce the anxiolytic effect of OT. It appears that the upregulation or downregulation of OT signaling by HPA axis activity not only depends on the brain region, but also on the duration of the stressor (acute vs. chronic) (Neumann, 2002). Hence, the neuroendocrine pathways causing variations in thermal maps and reduced OT levels may contribute to sex disparities in response to the presence of a male experimenter *via* a close interaction with the HPA axis (Liberzon and Young, 1997).

The present study showed inhomogeneous stress responses toward T-shirts worn by a male experimenter. A number of possibilities should be considered as the main source of discrepancy between the present results and earlier findings (Sorge et al., 2014). First, the T-shirts worn by males may only mimic the presence of an experimenter when specific neurophysiological measures such as pain behaviors and analgesic changes are addressed. Second, olfactory stimuli (e.g., axillary secretions) may differently affect female and male rats and male-associated olfactory inputs (Bateson, 2014) to induce greater alterations in females when combined with visual stimuli. Thus, stress-related arousal and physiology in female rats may be influenced by olfactory cues of male perspiration (Sorge et al., 2014), by visual cues of a present threat (Faraji et al., 2020) or by other neurohormonal stimuli (Herman and Cullinan, 1997; Ramos and Mormède, 1998).

An important corollary to repeated exposure to a male experimenter is the development of habituation, a normal pattern that indicates gradual coping with the stress-induced homeostatic disruption (Faraday, 2002). In contrast, a failure to habituate typically represents greater stress vulnerability. The impact of a male’s presence on experimental outcomes in the current study did not diminish over time, although animals were handled by the same- or opposite-sex experimenters for 10 days before and during the experiments. Accordingly, the disparate responses to male experimenters seen in female and male rats were maintained for the full 14 days of repeated exposure. This observation negates the potential that repeated exposure to a male experimenter prior to an experimental manipulation may induce stress tolerance or habituation (Faraday, 2002). However, the elevated levels of CORT in females in the first two experiments of this study do support an alternative hypothesis that HPA axis response, in specific conditions, fails to habituate to continuous stressor exposure (Schommer et al., 2003; Faraji et al., 2011, 2013).

## Conclusion and synthesis

The present study demonstrates that female and male experimenters represent a critical variable in rodent models, with female rats being more susceptible than males to the male observer effect. Moreover, the confounding effect of the male experimenter neither diminishes overtime, nor can it be merely attributed to human olfactory stimuli. We showed that the presence of a male experimenter activates the HPA axis with potentially wide-ranging effects on behavioural and physiological outcomes. The findings confirm earlier reports of females being more vulnerable than males in terms of endogenous OT secretion in response to social stimuli (Faraji et al., 2018a). Notably, the opposite-sex dynamics that has been recently reviewed in humans (Chapman et al., 2018), may also be applicable to animals in preclinical studies. However, three limitations of the present study should be acknowledged. First, the potential interference of locomotor activity with the IR thermal responses. The present strategy for randomization of sample selection and group assignment might diminish confounding effects of locomotion on thermal responses. However, conclusions about thermal responses in the absence of a proper control for the activity level in animals should be drawn with caution and warrant further consideration. Second, the baseline (pre-stress) levels of CORT were not determined. The present study follows an experimental design which controls confounding variable(s) that may impact the experimenter’s sex effect. Although there is potential for exposure to further procedural stress, the pre-stress measurement of CORT values is still suggested if variations in stress response are specifically considered in future investigations. Finally, the lack of an experimental group (no experimenter/no T-shirt) to control for the effect of an “experimenter.” It is difficult to avoid the presence of a human in the work with laboratory animals and their husbandry, and potentially lingering effects of stress 44 caused by handling preclude unambiguous assessment of an experimenter effect. Since the close interaction between the subject and experimenter impacts experimental outcomes, a hands-off test system that collects data throughout day and night in absence of a human experimenter may have the capacity to minimize the experimenter sex effect (Bohlen et al., 2014). Thus, the idea of a virtual experimenter (Horing et al., 2016, 2020; Chapman et al., 2018) that conceptualizes the application of an automated protocol and computer-based treatment in experiments, may pave the way to standardize experimental procedures and reduce artifacts in preclinical findings.

In line with an earlier report (Sorge et al., 2014), the present data are thought to encourage committed efforts to solve the replication crisis in the life sciences. While recent efforts have strengthened the consideration of sex differences in preclinical and clinical research, the present data suggest that also the experimenter’s biological sex represents a concern. Because experimenter sex in animal studies is not consistently reported in the scientific literature, improved standards should require researchers to report the sex of experimenters. Our results also indicate that repeated exposure to a female or male experimenter differently impacts female and male rats, and that both sexes are vulnerable to the disruptive effect of the mild stress when responding to human male experimenters in a sex-specific manner. The present findings therefore emphasize the critical importance of including female animals in preclinical research to address potential sex differences within a translational research framework (Jadavji and Metz, 2008; Beery, 2018; Faraji et al., 2018b).

## Data availability statement

The raw data supporting the conclusions of this article will be made available by the authors, without undue reservation.

## Ethics statement

The animal study was reviewed and approved by The University of Lethbridge Animal Care Committee in compliance with the standards set out by the Canadian Council for Animal Care (CCAC).

## Author contributions

JF and GM designed the study. JF, MA, NS, JT, KW, and NL performed the experiments and analyzed the data. JF wrote the manuscript. MA, NL, DO, and GM edited the manuscript. DO and GM acquired the funding and provided resources. All authors contributed to the article and approved the submitted version.

## Conflict of interest

The authors declare that the research was conducted in the absence of any commercial or financial relationships that could be construed as a potential conflict of interest.

## Publisher’s note

All claims expressed in this article are solely those of the authors and do not necessarily represent those of their affiliated organizations, or those of the publisher, the editors and the reviewers. Any product that may be evaluated in this article, or claim that may be made by its manufacturer, is not guaranteed or endorsed by the publisher.
