# Supplementary material for: Sex-specific stress and biobehavioral responses to human experimenters in rats
Source: Front Neurosci. 2022 Jul 22;16:965500. doi: 10.3389/fnins.2022.965500 (PMC9354940; doi:10.3389/fnins.2022.965500)
Supplement: Supplementary file 1 [file Data_Sheet_1.PDF]

(Supplemental Information)

# Sex-specific Stress and Biobehavioural Responses to Human Experimenters in Rats

Faraji *et al.*

## Supplemental Figures

**Figure 1.** *Correlational analysis*. Most trials in Experiment 1 to 4 revealed no significant correlation between surface temperature and thigmotaxis before and after stress (panels A&B). Stress-induced thermal changes in general did not predict post-stress thermal responses to the experimenter sex in both groups (panel C). Yellow, green and purple triangles in the corners represent *B. Baseline, C. Baseline*, and *D. IR recording (stress-open field)*, respectively. *$p \leq 0.05$, **$p \leq 0.01$; Pearson's correlation, n = 5-6/group.

**Figure 2.** *Correlational analysis*. (A) Plasma CORT and thigmotaxis were positively correlated in both sexes, particularly in female rats. (B) There was no significant correlation between the cutaneous temperature and CORT. (C) A significant negative correlation was found between the plasma CORT and OT in female rats in Experiments 1 and 2, whereas the correlation between the CORT and OT in male rats was not significant. Purple and pink triangles in the corners

represent *D. Stress* and *E. Blood Collection*, respectively. $^{**}p \leq 0.01$; Spearman correlation, n = 4-6/group.
