# Supplementary figures and images for: Sex-specific stress and biobehavioral responses to human experimenters in rats
Source: Front Neurosci. 2022 Jul 22;16:965500. doi: 10.3389/fnins.2022.965500 (PMC9354940; doi:10.3389/fnins.2022.965500)

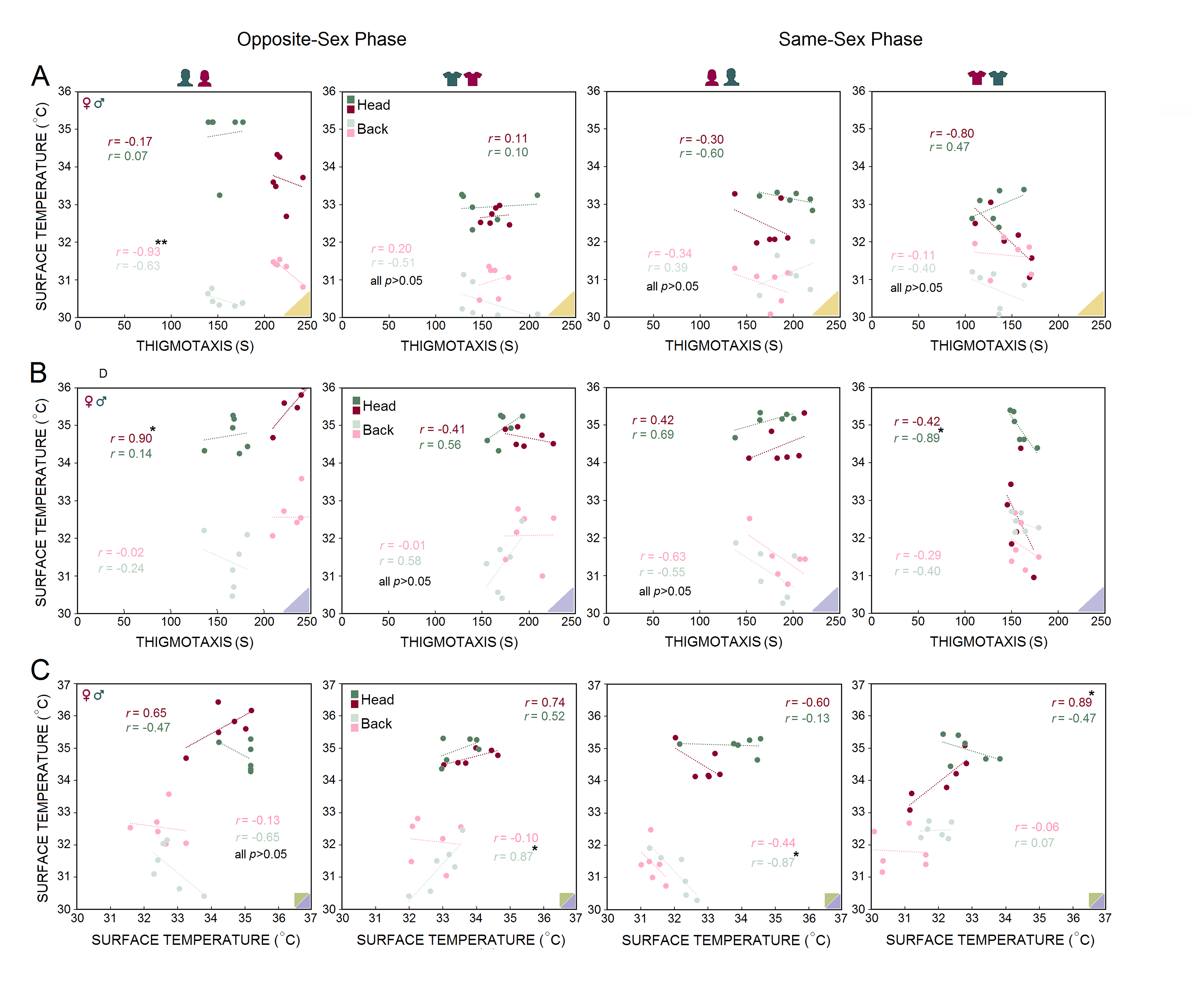

Supplement: Supplementary file 2 [file Image_1.TIF]

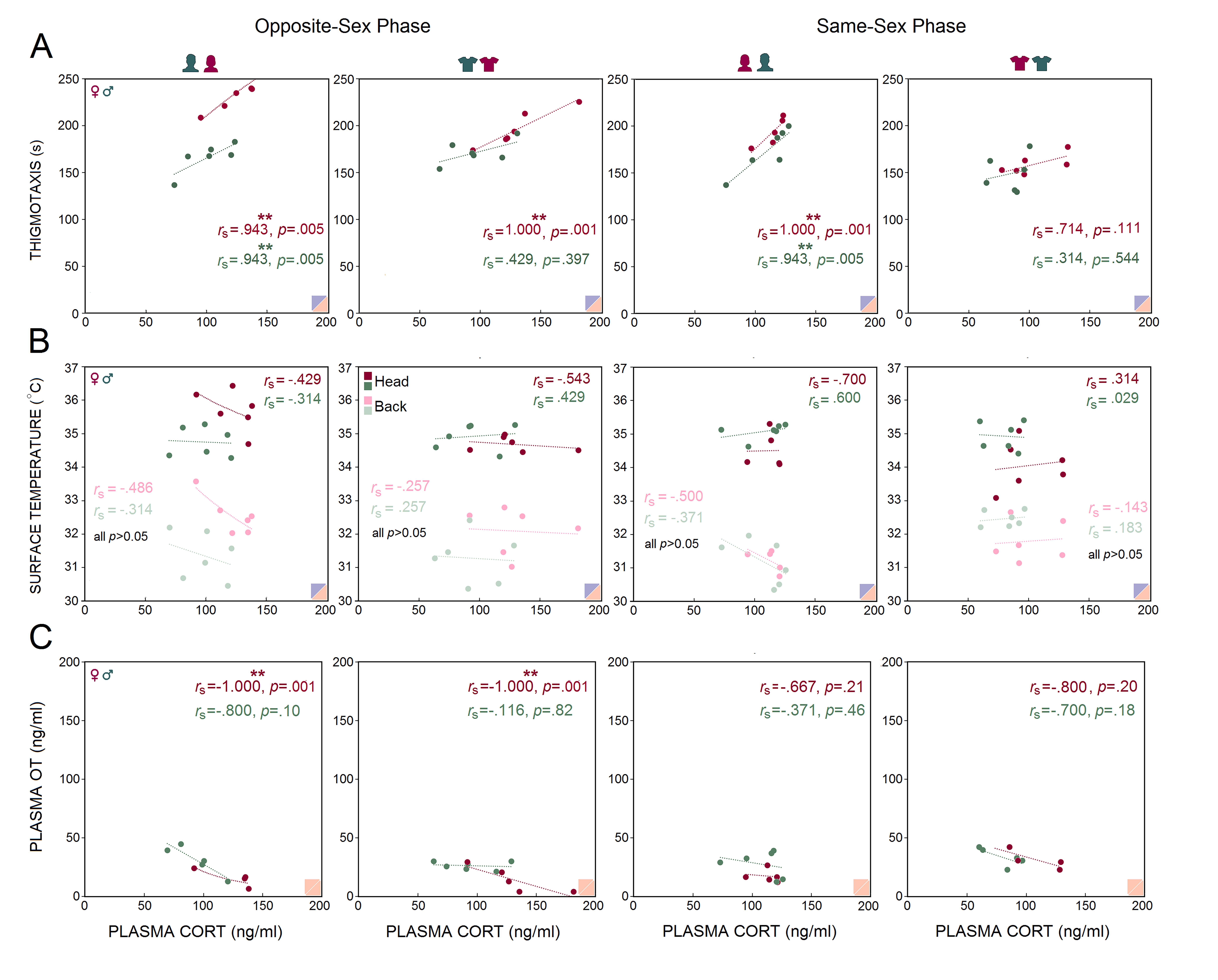

Supplement: Supplementary file 3 [file Image_2.TIF]
